# Increased serum methylmalonic acid levels were associated with the presence of cognitive dysfunction in older chronic kidney disease patients with albuminuria

**DOI:** 10.1186/s12877-024-04759-y

**Published:** 2024-02-15

**Authors:** Jialing Zhang, Leiyun Wu, Shiyuan Wang, Yajing Pan, Aihua Zhang

**Affiliations:** 1 Department of Nephrology, Xuanwu Hospital, Capital Medical University, Changchun Street 45#, 100053 Beijing, China; 2 National Clinical Research Center for Geriatric Disorders, Xuanwu Hospital, Capital Medical University, Beijing, China

**Keywords:** Albuminuria, Chronic kidney disease, Cognition impairment, Methylmalonic acid, Older adults

## Abstract

**Background:**

This study aimed to evaluate the correlation between serum methylmalonic acid (MMA) levels and cognition function in patients with chronic kidney disease (CKD).

**Methods:**

In this cross-sectional study, we included 537 CKD individuals aged ≥ 60-year-old with albuminuria from the National Health and Nutrition Examination Survey (NHANES) 2011–2014. Four cognitive tests including the Digit Symbol Substitution Test (DSST), the Consortium to Establish a Registry for Alzheimer’s Disease (CERAD) Delayed Recall and Word Learning tests, and the Animal Fluency test (AF) were performed. Associations between MMA and cognition scores were assessed with linear regression models.

**Results:**

MMA level was negatively associated with residual renal function and nutrition status. After multivariate adjustment, elevated serum MMA levels were independently correlated with decline of cognition in CKD patients with albuminuria.

**Conclusion:**

Our study showed that higher serum MMA levels were independently associated with the presence of cognition dysfunction in CKD patients. The exact pathogenesis of MMA and cognition needs further research.

## Introduction

Chronic kidney disease (CKD) is one of the most common global causes of morbidity and mortality [1]. Cognitive impairment is commonly found in patients with CKD. A recent systematic review yielded the prevalence of cognitive decline is more common in patients with CKD compared with patients without CKD [2]. Poor cognitive function has been linked to an increasing social and financial costs [3] and mortality [4]. The underlying pathophysiology of CKD-associated cognitive dysfunction is complex, including genes, uremia toxins, vascular dysfunction, and neuroinflammation [5].

Vitamin B12 is essential to regulate DNA synthesis, methylation reactions, and genomic stability. Low vitamin B12 level is associated with neurodegenerative disease, such as Alzheimer’s disease, vascular dementia, and Parkinson’s disease [6, 7]. Metabolic vitamin B12 deficiency is common, and it is necessary to measure functional markers of vitamin B12 adequacy such as methylmalonic acid (MMA) or homocysteine [8]. MMA is probably a specific and sensitive biomarker of subclinical vitamin B12 deficiency [9, 10]. An increase in serum MMA could be caused by decreased kidney function [11]. Serum MMA may be a favorable marker to predict various chronic diseases, including cardiovascular diseases [12, 13], and neurodegenerative diseases [14]. MMA is even more strongly associated with poor cognition and physical performance than serum vitamin B12 [15, 16]. Current evidence showed a relation between serum MMA, vitamin B12 status and cognitive function in older populations [17–19]. However, few studies have examined the role of MMA and cognitive decline in CKD patients with albuminuria.

Therefore, in this study, we aimed to explore whether elevated serum MMA levels are related to increased risk of cognitive impairment in patients with CKD.

## Methods

### Data source and participants

NHANES is a nationally representative, cross-sectional survey of the noninstitutionalized US population. The protocols for conducting the NHANES were approved by the institutional review board of the National Center for Health Statistics, Centers for Disease Control and Prevention, and informed consent was obtained from all participants.

Estimated GFR (eGFR) was calculated according to the Modification of Diet in Renal Disease (MDRD) calculation [20]. CKD was defined as: CKD stages G1–G3 (eGFR 30–59 mL/min/1.73 m2 or eGFR ≥ 60 mL/min/1.73 m2 and urea albumin/creatinine ratio (UACR) ≥ 30 mg/g) and CKD stages G4 and G5 (eGFR < 30 mL/min/1.73 m2), in accordance with the Kidney Disease: Improving Global Outcome 2012 Practice Guideline for CKD [21].

**Fig. 1:**
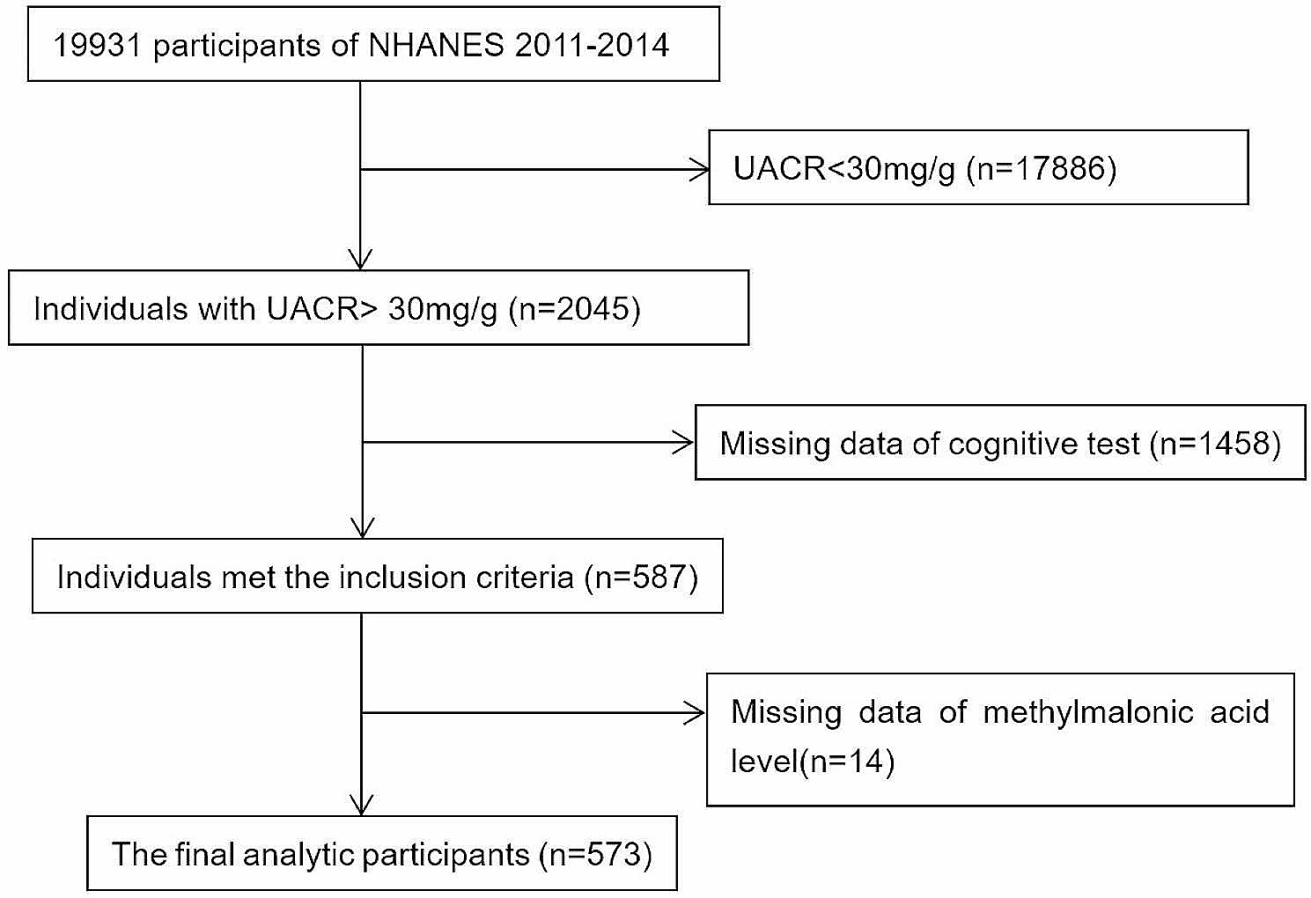
Flowchart of the study

For this study, we combined data from NHANES 2011–2014. A total of 2045 CKD patients with UACR > 30 mg/g aged 60 y and older participated in the NHANES health examination. The exclusion criteria were cases with missing key data. Ultimately, 573 participants were included in this study, see Fig. 1 for detail.

### Cognitive function assessment

Four separate tests formed the cognitive battery and were collected from the study participant. The Consortium to Establish a Registry for Alzheimer’s Disease (CERAD) is a comprehensive set of tests used to identify Alzheimer disease by assessing the ability for new learning, delayed recall, and recognition memory [22]. The CERAD-Word Learning test (CERAD-WL) consists of 3 consecutive learning trials, and the CERAD-Delayed Recall test (CERAD-DR) occurred after the other 2 cognitive exercises were completed. The Animal Fluency (AF) test asked participants to name as many animals as they can in 1 min to assess verbal fluency [23]. The Digit Symbol Substitution Test (DSST) is designed to measure processing speed, sustained attention, and working memory [24]. According to previous study, we used the lowest 25th percentile of the scores as the cutoff points to indicate cognitive impairment [25].

### Measurement of serum methylmalonic acid

Concentrations of MMA were measured in NHANES by the Division of Laboratory Sciences, National Center for Environmental Health, Centers for Disease Control and Prevention, Atlanta, GA. Detailed instructions on specimen collection and processing are discussed in the NHANES Laboratory Procedures Manual [26].

### Evaluation of covariates

Information on age, education level, sex, disease status, smoking status, and drinking status was obtained using questionnaires. In addition, hemoglobin, red blood cell distribution, serum vitamin B12, folate, total cholesterol, triglyceride, albumin, uric acid, vitamin D3, blood urea nitrogen (BUN), and creatinine levels were measured at baseline. Anemia is defined as hemoglobin level < 13.0 g/dl in males and < 12.0 g/dl in females [27]. The nutritional status was assessed by geriatric nutritional risk index (GNRI), which is relatively objective and effective [28]. Major nutrition-related risk is defined as GNRI < 82; moderate nutrition-related risk is defined as 82–92; low nutrition-related risk is defined as 92–98; and no risk is defined as > 98.

### Statistical analysis

We performed all statistical analyses using IBM SPSS Statistics 23. Patients were divided into four groups according to quartile of serum MMA. Categorical demographic variables were presented as numbers (weighted percentage) and compared among groups using Chi-square tests. We log-transformed MMA to normalize their distributions before statistical analysis. Relationships between MMA and covariates were examined through Spearman’s correlation analysis. Multivariable linear regression models were employed to explore the associations between MMA and each cognitive test. We adjusted for the covariates of age, sex, education level, smoke, drink, comorbidities, GNRI score, hemoglobin, albumin, cholesterol, uric acid, renal dysfunction, vitamin D3 and vitamin B12. In addition, subgroup analysis was also performed by linear regression analysis. Finally, receiver operating characteristic (ROC) curve was plotted and the area under the curve (AUC) was calculated. Two-sided *p*-values < 0.05 denoted significance.

## Results

### Baseline characteristics of the participants by the quartile of MMA levels

Table 1 presents the characteristics of the patients included in the study. A total of 573 CKD patients with albuminuria were included in the final data analysis. Patients with elevated MMA levels were likely to be older, with a lower hemoglobin, albumin, GNRI score, vitamin B12, and higher BUN, creatinine, and uric acid level. Besides, the proportion of hypertension, diabetes and anemia significantly differed among four groups. As for each cognitive test, patients in the group 4 had a relatively lower score of CERAD-WL, AF test, and DSST.

**Table 1:** Baseline characteristics of the participants by the quartile of MMA levels

	Total (*n* = 573)	Quartile 1 (*n* = 143)	Quartile 2 (*n* = 146)	Quartile 3 (*n* = 142)	Quartile 4 (*n* = 142)	*P*
Age (year)	71 (65–80)	67 (63–72)	72 (64.75-80)	73 (66–80)	74 (67.75-80)	< 0.001
Male (%)	52.4	53.8	54.1	52.1	49.3	0.259
Education						
Less than 9th grade (%)	16.4	16.8	16.4	14.8	17.6	
9 − 11th grade (%)	17.1	14	17.1	19	18.3	
High school graduate (%)	24.6	26.6	23.3	22.5	26.1	
College or AA degree (%)	25	25.9	24	25.4	24.6	
College graduate or above (%)	16.9	16.8	19.2	18.3	13.4	
Smoke (%)	37	40.7	34.5	36	36.6	< 0.001
Drink (%)	64.2	69.6	60.6	62.5	64.2	< 0.001
Hypertension (%)	75.3	74.8	78.1	70.4	77.5	< 0.001
Diabetes (%)	41.2	44.1	39	40.1	40.8	< 0.001
BMI (kg/m2)	28.12 (24.25–32.65)	28.05 (24.75–32.3)	28.66 (25.41–33.29)	27.59 (23.6-32.47)	28.17 (24.38–32.5)	0.295
GNRI	104.18 (100.1-107.29)	105.53 (101.87-108.54)	105.4 (102.66-107.43)	104.32 (100.87-107.31)	103.34 (99.72-106.98)	0.006
< 82 (%)	10.5	2.1	3.4	4.2	4.9	
82–92 (%)	1.1		0.7	2.8	1.4	
92–98 (%)	5.8	6.3	2.7	4.2	12	
> 98 (%)	82.5	91.6	93.2	88.7	81.7	
Hemoglobin (g/dl)	13.5 (12.5–14.5)	13.7 (13-14.7)	13.61 ± 1.42	13.45 ± 1.54	13.09 ± 1.82	0.001
Anemia (%)	10.5	4.2	7.5	12	17.6	0.001
RDW (%)	13.7 (13-14.6)	13.5 (12.8–14.3)	13.6 (13-14.4)	13.6 (13-14.53)	14.05 (13.3–14.9)	0.002
Albumin (g/l)	42 (39–43)	42 (40–44)	42 (40–43)	41 (39–43)	41 (38–43)	0.013
Cholesterol (mmol/l)	4.66 (3.91–5.61)	4.68 (3.83–5.82)	4.62 (3.85–5.72)	4.66 (4.01–5.33)	4.79 ± 1.19	0.945
Triglyceride (mmol/l)	1.55 (1.04–2.43)	1.49 (0.85–2.41)	1.59 (1-2.39)	1.52 (1.11–2.44)	1.62 (1.12–2.44)	0.378
BUN (mmol/l)	11 (6.07-17)	8.57 (4.64-13)	9 (5.71-16)	12 (7.5–19.5)	15 (8.48-25)	< 0.001
Creatinine (mmol/l)	90.17 (71.6-117.57)	74.26 (63.65–87.52)	89.73 (72.49-108.95)	99.89 (79.12-123.76)	110.06 (81.11-179.23)	< 0.001
Uric acid (mg/dl)	5.9 (4.9–7.2)	5.4 (4.6–6.6)	5.8 (5–7)	5.9 (5.1–7.2)	6.45 (4.9–7.8)	< 0.001
Vitamin D3 (nmol/l)	61.3 (41.64–85.05)	58.93 (39.7-77.94)	65.27 (41.72–85.45)	64.75 (46.15–88.78)	58.45 (38.9–89.9)	0.178
Vitamin B12 (pg/ml)	556 (386–775)	638 (403.5–850)	617 (462–812)	552.5 (384-780.25)	462 (318.5–643)	< 0.001
Folate (ng/ml)	549.5 (404–787)	498.5 (369.75-671.25)	596 (415–848)	589.5 (415.5–799)	556 (422.5–830)	0.021
CERAD-WL	18 (14–21)	19 (14–22)	18 (14.75-21)	17 (14–21)	16.5 (13–20)	0.022
CERAD-DR	6 (4–7)	6 (4–7)	6 (4–7)	5 (3–7)	5 (3–7)	0.181
Animal frequency test	15 (11–18)	15.31 ± 5.35	15 (11.75-18)	15 (11–19)	14 (10–18)	0.047
DSST	38 (24–49)	38.97 ± 18.12	39.36 ± 18.18	35.73 ± 19.41	32 (19.75-44)	0.001
Methylmalonic acid	194 (141.5–283)	115 (100–129)	168 (153–183)	221.5 (204-244.25)	397.5 (329.5-536.5)	< 0.001

AF, Animal Fluency test; CERAD-WL, Consortium to Establish a Registry for Alzheimer’s Disease Word Learning test; CERAD-DR, Consortium to Establish a Registry for Alzheimer’s Disease Delayed Recall test; DSST, Digit Symbol Substitution test; CKD, chronic kidney disease; BMI, body mass index; GNRI, geriatric nutritional risk index; RDW, red blood cell distribution width; BUN, blood urea nitrogen

### Relations of MMA with covariates

The association of MMA with other covariates was shown in Table 2. In Spearman’s correlation analysis, serum MMA level was positively associated with age, BUN, creatinine, UACR, folate, and uric acid, while negative associations were found between serum MMA leves and hemoglobin, albumin, eGFR, GNRI, and vitamin B12.

**Table 2:** Associated factors of MMA levels

	ρ	*P*
Age (year)	0.272	< 0.001
BMI (kg/m2)	-0.055	0.185
GNRI	-0.138	0.001
Hemoglobin (g/dl)	-0.164	< 0.001
Albumin (g/l)	-0.124	0.003
Cholesterol (mmol/l)	-0.01	0.814
Triglyceride (mmol/l)	0.067	0.11
BUN (mmol/l)	0.297	< 0.001
Creatinine (mmol/l)	0.413	< 0.001
eGFR (ml/min/1.73m^2^)	-0.466	< 0.001
UACR (mg/g)	0.146	< 0.001
Uric acid (mg/dl)	0.189	< 0.001
Vitamin D3 (nmol/l)	0.033	0.432
Vitamin B12 (pg/ml)	-0.228	< 0.001
Folate (ng/ml)	0.098	0.019
CERAD-WL	-0.114	0.006
CERAD-DR	-0.088	0.035
AF test	-0.105	0.012
DSST	-0.149	< 0.001

AF, Animal Fluency; CERAD-WL, Consortium to Establish a Registry for Alzheimer’s Disease Word Learning test; CERAD-DR, Consortium to Establish a Registry for Alzheimer’s Disease Delayed Recall test; DSST, Digit Symbol Substitution test; BMI, body mass index; GNRI, geriatric nutritional risk index; BUN, blood urea nitrogen; UACR, urea albumin/creatinine ratio; eGFR, estimated glomerular filtration rate

### Relations of MMA with cognitive dysfunction

In our Spearman’s correlation analysis, the score of CERAD-WL, CERAD-DR, AF test, and DSST were all negatively associated with serum MMA concentrations. Furthermore, after adjusting for other confounding factors, the standardized β of MMA for CERAD-WL and AF test was − 2.191 (95%CI: -4.136, -0.246) and − 1.992 (95%CI: -3.913, -0.072), respectively. We found that an increasing level of MMA was probably an independent associated factor for a declining of cognitive function in patients with CKD (shown in Table 3).

**Table 3:** Multivariable linear regression for the association between MMA levels and cognition function in older patients with CKD

	Standardized β (95% CI)	*P*
CERAD-WL	-2.191 (-4.136, -0.246)	0.027
CERAD-DR	-0.177 (-1.026, 0.672)	0.683
AF test	-1.992 (-3.913, -0.072)	0.042
DSST	-2.759 (-8.321, 2.803)	0.33

AF, Animal Fluency; CERAD-WL, Consortium to Establish a Registry for Alzheimer’s Disease Word Learning test; CERAD-DR, Consortium to Establish a Registry for Alzheimer’s Disease Delayed Recall test; DSST, Digit Symbol Substitution test

### Stratification analyses

We performed subgroup analyses and analyzed the interactions between MMA and the variables in this study (shown in Table 4). In the stratified analyses, we divided patients into subgroups according to sex, comorbidities, level of UACR, nutritional status and median value of vitamin B12. We found that the subgroups of male sex, and non-hypertension were similar to our main results. Namely, MMA negatively correlated with CERAD-WL and AF scores in male and non-hypertension patients. Besides, serum MMA was negatively associated with CERAD-WL in patients with diabetes, a relatively lower level of vitamin B12 and a higher GNRI score. MMA was also an independent associated factor for AF test in patients with malnutrition and macroalbuminuria. However, there was no significant relationship between each cognitive test and serum MMA level in the subgroups of female sex, hypertension, non-diabetes, and a higher level of vitamin B12. The results also implied that the effect of MMA on cognition function in CKD patients could be affected by URCR levels (P for interaction < 0.05). In contrast, hypertension, diabetes, vitamin B12 and nutritional status did not modify the associations between MMA and cognition function (p for interaction > 0.05).

**Table 4:** Stratified analyses of the associations between serum MMA concentrations and cognitive function among older patients with CKD

	CERAD-WL			AF test		
	Standardized β, 95% confidence intervals	*P*	*P* for interaction	Standardized β, 95% confidence intervals	*P*	*P* for interaction
Sex			0.622			0.039
Male	-2.593 (-4.921, -0.265)	0.029		-2,73 (-5.458, -0.001)	0.05	
Female	-2.456 (-5.942, 1.029)	0.166		-0.825 (-3.848, 2.198)	0.592	
Hypertension			0.213			0.809
Yes	-0.983 (-3.308, 1.341)	0.406		-0.063 (-2.381, 2.255)	0.958	
No	-4.809 (-8.726, -0.892)	0.017		-6.996 (-10.847, -3.145)	< 0.001	
Diabetes			0.059			0.368
Yes	-5.092 (-8.871, -1.313)	0.009		-3.406 (-6.983, 0.171)	0.062	
No	-1.278 (-3.61, 1.053)	0.281		-1.566 (-3.96, 0.828)	0.199	
UACR level			0.01			< 0.001
30 mg/g < UACR < 300 mg/g	-1.881 (-4.193, 0.431)	0.111		-0.366 (-2.67, 1.938)	0.755	
UACR > 300 mg/g	-2.733 (-7.31, 1.845)	0.238		-6.858 (-10.875, -2.842)	0.001	
Vitamin B12			0.934			0.562
< 556 pg/ml	-3.129 (-5.742, -0.516)	0.019		-2.15 (-4.775, 0.475)	0.108	
> 556 pg/ml	-0.083 (-3.723, 3.558)	0.964		0.061 (-4.074, 2.854)	0.729	
GNRI			0.541			0.104
< 98	4.564 (-2.144, 11.272)	0.176		-8.62 (-14.399, -2.841)	0.004	
> 98	-3.919 (-6.047, -1.791)	< 0.001		-1.452 (-3.739, 0.561)	0.147	

AF, Animal Fluency; CERAD-WL, Consortium to Establish a Registry for Alzheimer’s Disease Word Learning test; UACR, urea albumin/creatinine ratio; GNRI, geriatric nutritional risk index

### The diagnostic value of MMA for cognition function in CKD patients

The best cutoff for the ROC curve was calculated with the Youden’s index. The optimal point of MMA for cognitive impairment was 194.5nmol/l (AUC = 0.567, sensitivity 57%, specificity 56%, *P* = 0.006) as measured by CERAD-WL and 181.5nmol/l (AUC = 0.565, sensitivity 65%, specificity 53%, *P* = 0.015) as measured by AFT, respectively.

## Discussion

The final analysis results of this study showed that the increase of MMA concentration was significantly related to the decline of cognitive level in CKD patients with albuminuria. Furthermore, serum MMA level was negatively related to cognitive test score in CKD patients of male sex, patients without hypertension, and absence of vitamin B12.

CKD is defined by indicators of kidney damage—imaging or proteinuria (i.e.,UACR)—and decreased renal function for at least three months [29]. The burden of cognitive impairments in CKD has been extensively studied [30, 31], with prevalence ranging from 13–58% [32–34]. Cognitive impairment is an independent predictor of mortality and morbidity in end-stage renal disease patients [4, 35].

Disruption of vitamin B12 transport in the blood, or impaired cellular uptake might cause an intracellular deficiency. Diagnostic biomarkers for vitamin B12 status include decreased levels of circulating total vitamin B12, and abnormally increased levels of homocysteine and MMA. The metabolism of MMA in mitochondria could be hindered by mitochondrial methylmalonyl-CoA mutase deactivation or coenzyme active vitamin B12 deficiency, leading to an accumulating of MMA [36]. Some studies suggested that the increase of MMA was related to cognition impairment in children aged 3–16 [37], and participants aged 61–87 [38, 39]. However, a previous meta-analysis included 11 studies, and performed no correlation between the increase of MMA level and the decrease of cognitive level in the general population [40]. It is essential to definite whether a higher level of MMA might lead to cognitive decline in patients with CKD.

In our study, we included CKD patients with UACR > 30 mg/g to determine the relationship between MMA and cognitive dysfunction. We found that serum MMA level was negatively associated with cognitive score in CKD patients with albuminuria. Previous study found that impaired kidney function could increase MMA [41]. The renal disease of MMA could be induced by proximal renal tubular mitochondrial dysfunction [42]. In our study, we also found that serum MMA was positively associated with BUN, creatinine and proteinuria level, and negatively associated with eGFR. Our findings indicated that an accumulating in MMA perhaps due to a decline of renal function. Furthermore, the association between MMA and cognition dysfunction tended to be stronger in patients with macroalbuminuria in comparison to patients with microalbuminuria. Circulating level of MMA is strongly associated with elevated all-cause and cardiovascular mortality in adults [43]. Multiple survey determined MMA was inversely associated with cognitive function scores in the elderly general population [44–46]. The mechanisms responsible for the neurological dysfunction in methylmalonic acidemia have so far not been fully elucidated.

It is known that serum MMA levels increased with age [47]. MMA might have neurotoxicity, since its accumulating in cerebrospinal fluid [48]. A larger echogenic area of the substantia nigra was reported to be related to higher serum concentrations of MMA in Parkinson disease [49]. A study has investigated cross-sectional association between circulating MMA and brain volumes, although no significant association was observed in the fully adjusted model [50]. An elevated MMA could inhibit respiratory chain and impair energy metabolism in hippocampus tissue [51]. Furthermore, neurologic deficit in methylmalonic acidemia might be due to MMA-induced lipoperoxidation in cerebral cortex [52]. Depolarization of the plasma membrane and neuronal cell apoptosis caused by MMA might be another key mechanism [53, 54]. Serum uric acid was positively associated with MMA in this study, which was also found to be independently related to cognition dysfunction [16]. Evidence of mitochondrial reactive oxygen species generation and oxidative stress were suggested to contribute to the disorder [55]. An inverse association of albumin, hemoglobin, GNRI and MMA was observed in our study, probably indicating a poor nutrition status in patients with high MMA levels. Similar, malnutrition is also correlated with the deterioration cognitive domains [56].

In our subgroup analysis, we found MMA was an associated factor for cognitive impairment in patients with diabetes, but not patients without diabetes. Studies demonstrated that elevated MMA levels in diabetes patients acted as an indicator for peripheral neuropathy [57, 58]. Moreover, MMA accumulation was positively associated with increased mortality risk in type 2 diabetes patients [59]. Higher serum MMA was associated with the presence of cardiovascular diseases [13]. Plasma vitamin B12 was positively associated with hypertension in women [60]. In our study, we found the association of MMA and cognitive function was not significant in patients with hypertension. Hypertension has represented an important risk factor for cognitive decline, and a strict blood pressure control could prevent the progression [61, 62]. Another study explored the relation of the coexistence of high folate and low vitamin B12 status with cognitive function [63]. Having low vitamin B12/high folic acid status was associated with greater risk for cognitive impairment, when comparing to the high folate and normal vitamin B12 status. From our analysis, we did not find any significant association between MMA and cognitive score in the subgroup of high vitamin B12 group. It is reported that serum MMA and vitamin B12 concentrations do not directly correlate with each other [64]. The association of cognition with MMA is stronger than that with vitamin B12 [64]. Hence, a supplementation of vitamin B12 and ensuring optimal concentrations of both vitamin B12 and MMA might improve potential clinical prognosis. Since the predictive power of MMA for cognition deficit in CKD patients is relatively weak in this study, other longitudinal studies were needed to confirm our results.

Our study firstly demonstrated the association of MMA and cognition function in CKD patients. The use of comprehensive data from the NHANES allowed us to control for potential key confounders. This study had several limitations. First, this cross-sectional study could not establish a cause-effect association of MMA and cognition. Second, the sample size of this study was relatively small, which might lead to a bias. Third, self-reported variables and other unmeasured confounders could affect our results.

## Conclusion

To sum up, higher serum MMA were associated with the presence of cognition impairment in older CKD patients. Besides, serum MMA levels negatively correlated to nutrition status, and residual renal function. Our results highlight that MMA could be a therapeutic target for cognition dysfunction in CKD patients, and further studies are needed to clarify its mechanism.

## Data Availability

The datasets generated and analyzed in the present study are available on the website of NHANES datasets 2011–2014 (https://wwwn.cdc.gov/nchs/nhanes).
